# OFBG-Based Smart Double-Skin Tubular Confined-Concrete Column with Basalt FRP-Steel Composite

**DOI:** 10.3390/s19163572

**Published:** 2019-08-16

**Authors:** Yung William Sasy Chan, Zhi Zhou, Wanqiu Liu, Jinping Ou

**Affiliations:** 1 Department of Civil Engineering, Dalian University of Technology, Dalian 116024, China; 2 State Key Laboratory of Marine Resource Utilization in South China Sea, College of Civil Engineering and Architecture, Hainan University, Haikou 570228, China; 3 State Key Laboratory of Coastal and Offshore Engineering, Dalian University of Technology, Dalian 116024, China; 4 School of Civil Engineering, Harbin Institute of Technology, Harbin 150090, China

**Keywords:** self-sensing, double-skin tubular column, built-in OFBG, FRP-strengthened steel, punched-in pattern steel, life cycle strain monitoring

## Abstract

Fiber-reinforced polymer (FRP) composites have been widely employed to design advanced structural columns such as the hybrid FRP–concrete–steel double-skin tubular column (hybrid DSTC) with potential benefits. To date, the safety and self-monitoring of the hybrid DSTCs are still a challenge to overcome due to the complex damage scenarios. This paper investigates the self-sensing performance of a newly developed smart double-skin tubular confined concrete column (smart BFST-DSTC) made of basalt FRP–steel composite with built-in optical fiber Bragg grating sensors (OFBGs). The design of the smart BFST-DSTC and sensing principle are firstly addressed, followed by an experimental investigation on the basic mechanical properties and strain-based sensing performance of ten scaled specimens under axial compression. The outcomes reveal the enhancement of the proposed column in terms of load-carrying capacity, confinement ratio, and axial stress-axial strain behavior, as well as failure and damage modes when compared with the hybrid DSTC. The self-sensing investigation demonstrates that the measurement range satisfies the requirement to monitor and evaluate the hoop strains of the FRP jackets and the health state of the inner tube. The smart BFST-DSTC can replace the hybrid DSTC with the ability to provide early failure warning and life cycle health monitoring.

## 1. Introduction

Fiber-reinforced polymer (FRP) composites have been widely used as civil infrastructure construction materials in the world due to their high strength-to-weight ratio, good corrosion resistance, and tailorable mechanical properties. FRP composites offer ample possibilities for advanced structural columns including concrete-filled FRP tube (CFFT) [1], FRP-confined concrete-filled steel tube (CFST) [2], and hybrid FRP–concrete–steel double-skin tubular column (hybrid DSTC) [3]. The hybrid DSTC appears advantageous due to the good combination of the outer FRP tube, the inner steel tube, and the concrete in between. Despite presenting high-strength and good ductility properties under flexural [4] and axial compression [5,6,7,8], the accumulated deformation occurring in the hybrid DSTC leads to multiple damage scenarios before reaching its ultimate loading capacity. The progressive damage includes concrete fragmentation, buckling of the inner steel tube, fiber-matrix debonding, and sudden fiber failure of the outer FRP tube [9,10]. Consequently, it is important to design a stronger column with higher safety and a life cycle self-sensing ability.

Different methods have been developed for the damage detection in FRP-based confined tubular columns. Li et al. used acoustic emission (AE) technology to analyze the damage of CFST [11]. The severity of the damage has been estimated using the AE signals. However, AE technology can only give a response when damage has already happened, and it often requires complex data analysis. The piezoceramic (PZT)-based method has been used to detect the interfacial condition between the concrete and the confinement material of CFST [12] and CFFT [13]. The experimental findings showed that the embedded PZT sensors detect and locate the interface debonding between the concrete infill and the confinement material. However, the size of the PZT sensors limits its integration into the FRP tube. OFBGs have been installed in the concrete–FRP interface to measure the strain state of the concrete columns confined with FRP [14] and embedded in the inter-ply to measure the strain of the outer FRP tube in CFFT [15]. However, these studies [14,15] focused only on the strain measurement during the strengthening stage. They did not investigate the damage detection of the CFFT beyond the strengthening stage, and their study was limited to the strain monitoring of the outer FRP tube. Currently, there is no investigation on the health monitoring of the hybrid DSTC.

Optical fiber sensors (OFS) are easy to integrate into complex materials due to their tiny dimensions, lightweight size, low cost, and immunity to electromagnetic interference. Researchers have demonstrated the excellent properties of OFS packaged into FRP materials for strain and temperature change detection in civil infrastructures [16,17,18]. Based on this principle, some smart FRP-based components with built-in OFS have been developed to contribute to the life cycle monitoring of civil infrastructures from inception through to the construction and service stage until final decommissioning [19,20,21]. Li et al. also revealed that the strain sensitivity of OFBGs remains constant after embedding into glass FRP (GFRP) [22]. The built-in method with FRP reduces the cost of sensor installation and allows the monitoring of the structure where sensor installation is quasi-impossible [23,24]. The strain measurement range for embedded OFBGs in FRP has been enhanced up to 8000 με [25]. Zhou et al. [24] have verified that the built-in OFBG-FRP rebar can be well-combined with steel material to detect strain in the FRP–steel smart strand. On the other hand, basalt FRP (BFRP) presents some advantages compared to traditional carbon, glass, and aramid FRP. With the ultimate strain close to the GFRP, excellent environmental resistance, thermal expansion coefficient close to concrete, and low cost, BFRP is an excellent candidate for OFS packaging [26]. The benefits of BFRP allow designing smart components with wide strain range, long-term stability, reliable protection, a decrease of temperature stress, and low cost.

However, it is difficult to embed OFBGs in the inner steel tube of the hybrid DSTC. FRP-jacketed steel (FRP–steel composite) has been used in CFST to solve the corrosion issue and enhance the mechanical behavior of the column [27,28,29]. The FRP jacket delays the buckling of the steel tube, as well as enhances the ductility and failure mode of the column [30]. The FRP jacket can also delay the local buckling of deficient hollow steel columns [31] and the elephant’s foot buckling of cylinder shells [32]. However, the full utilization of the tensile strength from the outer FRP jacket is a critical issue to achieve high confinement efficiency [33]. 

This paper investigates the self-sensing performance of a newly developed OFBG-based smart DSTC with basalt FRP–steel composite (smart BFST-DSTC) under axial compression. The smart BFST-DSTC consists of inner and outer basalt FRP–steel composite tubes with built-in OFBGs. The designed manufacturing process and self-sensing principle are first highlighted. A series of axial compression tests on ten scaled specimens were conducted to investigate the basic mechanical properties and the strain-based self-sensing performance. Parameter designs (concrete strength, outer steel skin with punched-in pattern, thickness of steel skin, and FRP jackets layer number) that influence the strain-sensing properties were also studied briefly. The smart BFST-DSTC has potential mechanical benefits to overtake the hybrid DSTC as a modern form of structural column. The embedded OFBGs can monitor the strain developed in the column up to 70% of the strengthening load, which is wide enough to detect minor damage occurring in the BFST-DSTC. Finally, the wavelength shift (WS) from the inner FRP jacket allows us to evaluate damage beyond the ultimate load of the column. 

## 2. Design, Manufacturing, and Sensing Principle of the Smart BFST-DSTC

### 2.1. Design of the Smart BFST-DSTC

**Figure 1:**
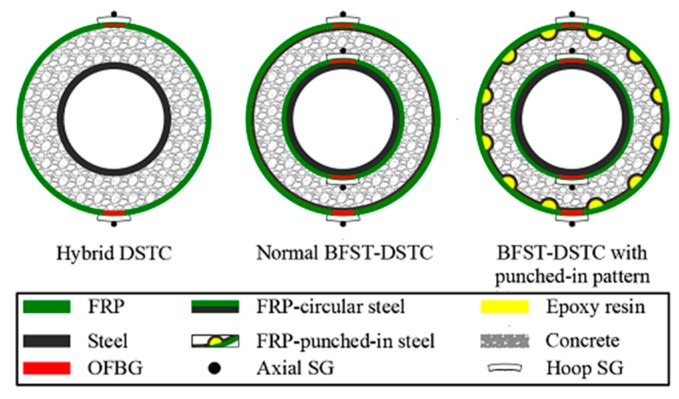
Cross-sectional design for hybrid FRP–concrete–steel double-skin tubular column (hybrid DSTC), normal BFST-DSTC, and BFST-DSTC with punched-in pattern.

The smart BFST-DSTC consists of an inner and outer self-sensing basalt FRP–steel tube (BFSTs), and concrete in between. The BFSTs with built-in OFBGs are manufactured first, before the production of the smart BFST-DSTC. Similar to the hybrid DSTC, the inner BFST provides longitudinal reinforcement, and the outer BFST plays a role as lateral confinement. Thus, the inner BFST has a thicker steel layer than the outer BFST. The use of FRP jacket in the inner BFST delays the plastic deformation and even the buckling of the steel. The OFBG sensors’ layout design is based on the preliminary analysis of the stress distribution and the monitoring purpose. For the typical damage modes of the DSTC, such as cracking and buckling, the damage evolution process is always accompanied by the continuous increase of the hoop strain. However, the strain variation characteristic is not consistent for the vertical strain under damage. Therefore, the OFBGs are only embedded in the hoop direction for both the inner and outer FRP jackets. To be more sensitive to the damage or failure, the OFBGs are positioned at the middle height of the column. The BFST has two OFBGs positioned at the middle height in the circumference direction between the FRP layers (red marker, see Figure 1).

### 2.2. Fabrication of the Scaled Smart BFST-DSTC

**Figure 2:**
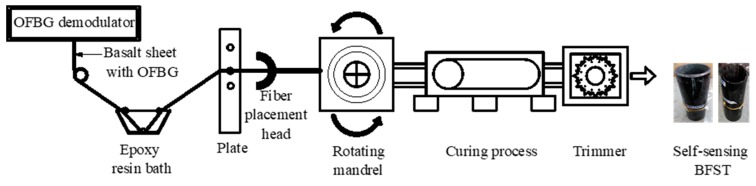
Manufacturing process for the self-sensing basalt fiber-reinforced polymer (FRP)–steel composite (BFST).

Embedding OFBGs into FRP-based structural component is advantageous for developing novel smart components. However, conventional OFBGs are fragile and could easily break during the wrapping process. Basalt fibers are used as a sheath to protect the OFBGs. Figure 2 shows the detailed manufacturing process using the filament winding/wrapping method. For the BFST tube, the steel serves as a permanent mandrel on the wrapping tool. The basalt sheet with OFBGs is first dipped in the epoxy resin bath for shaping. Then it is passed through a plate to control the dosage of the epoxy resin before wrapping around the steel. A fiber placement head guides the optical fiber in the basalt sheet to the desired position. During the wrapping process, all the OFBGs are connected to an OFBGs demodulator to monitor the production quality continuously. The composite tube is cured for a specific time to reduce the temperature before entering the trimmer. After the trimming process, the final self-sensing BFST is obtained. The scaled BFST-DSTC test sample is then fabricated by filling concrete in-between the BFSTs.

### 2.3. Sensing Principle of the OFBG

The Bragg gratings are formed by exposing the core of the optical fiber to the UV-laser light. The refractive index of the gratings section is different from that of the optical fiber. The formed grating only reflects a specified wavelength that is related to its grating period when a broadband source of light travels in the fiber. The reflected wavelength, known as the Bragg wavelength (*λ*_B_), is expressed in Equation (1) [15].

(1) $$\lambda_{B}=2n_{eff}\Lambda$$

where *n*_eff_ represents the effective refractive index and Λ is the grating period of the optical fiber. 

The Bragg wavelength (*λ*_B_) varies when a load is applied to the fiber. A stretching or tension loading results in a positive wavelength shift (Δ*λ*_B_), while a compression force produces a negative shift. However, the Bragg wavelength shifts (WS) are linearly proportional to the applied strain and temperature variation. The cross-sensitivity of the Bragg WS can be expressed in Equation (2) [15].

(2) $$\Delta \lambda_{B}(\Delta \varepsilon ,\Delta T)=\alpha_{\varepsilon }\Delta \varepsilon +\alpha_{T}\Delta T$$

where *α*_ε_ and *α*_T_ are the strain and temperature sensitivity coefficients of the OFBGs, respectively. 

In a laboratory test with constant temperature, the WS varies with the applied strain, given in Equation (3).

(3) $$\Delta \lambda_{B}(\Delta \varepsilon ,0)=\alpha_{\varepsilon }\Delta \varepsilon .$$

The WS of a free-strain OFBG can be expressed in Equation (4).

(4) $$\Delta \lambda_{B}(0,\Delta T)=\alpha_{T}\Delta T.$$

Thus, the relative applied strain is obtained by Equation (5).

(5) $$\Delta \varepsilon =\left[\Delta \lambda_{B}(\Delta \varepsilon ,\Delta T)-\Delta \lambda_{B}(0,\Delta T)\right]/\alpha_{\varepsilon }.$$

## 3. Lab Tests on the Smart Columns

### 3.1. Test Specimens

Ten specimens, including two hybrid DSTCs and eight BFST-DSTCs with different structural designs, were tested for comparison. The eight BFST-DSTCs included two normal BFST-DSTCs and six BFST-DSTCs with punched-in patterns. All of the test specimens were designed based on the guidelines in GB50608-2010 [34]. The specimens were manufactured based on the fabrication processes described in Section 2.2. The radial punched-in pattern of the outer steel was selected to increase the confinement effectiveness and reduce the brittle failure risk of the outer FRP jacket. Figure 1 shows the cross-sectional design for the hybrid DSTC, the normal BFST-DSTC, and the BFST-DSTC with punched-in pattern. The number of FRP sheet layers and the steel thickness were considered as variable parameters according to the demand in construction (Table 1). All the smart BFST-DSTCs were made with outer self-sensing BFST, whereas, only five had inner self-sensing BFST due to accidental damage of the optical fiber jumpers for specimens BFST-DSTC1, BFST-DSTC4, and BFST-DSTC6. The optical fiber jumpers for DSTC2 was also broken before test. Thus, the self-sensing performance of the DSTC2 is unavailable in the test results. A uniform inner steel tube thickness of 4 mm was chosen for all the scaled columns. The BFST-DSTC scaled columns had a different inner FRP jacket thickness. One specimen with full round outer steel for each concrete type was designed to compare with the corresponding specimen with punched-in pattern.

### 3.2. Materials

The experiment involved the use of self-compacted concrete, carbon steel, and unidirectional basalt fiber sheets. The concrete cylinder compressive strength (*f*_co_) could be converted by the concrete cubic strength obtained from the test at 28 days. The *f*_co_ were 20.02 MPa and 60.55 MPa for the normal strength concrete (NSC) and high-strength concrete (HSC), respectively. The steel used in the inner tube of all specimens consisted of galvanized Q275. The steel layer in the outer BFST consisted of a low-grade steel plate, where the radial punched-in patterns were manually formed. The tensile test samples were conducted by following the British standard BS18 [35] to provide the mechanical properties for each type of steel. Table 2 reports the mechanical properties of all steel materials. The unidirectional basalt fiber sheet had a nominal thickness of 0.115 mm. One layer and two layers of basalt FRP (BFRP) flat samples were tested according to the ASTM standard D3039 [36] to determine the tensile properties of the BFRP. The basalt fiber sheet used in the current experiment had an average tensile strength of 2030.43 MPa, a tensile modulus of 97.77 GPa, and an average rupture strain of 0.02115. 

### 3.3. Test Set-Up and Loading Procedure

**Figure 3:**
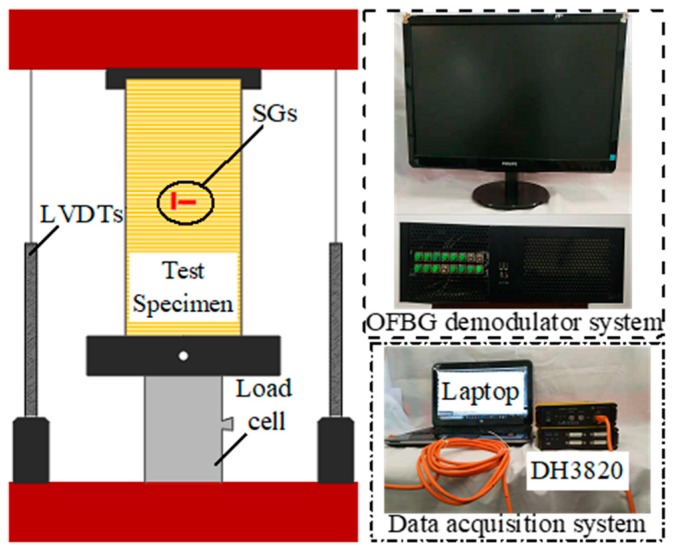
Test set-up.

Axial and hoop strain gauges (SG) were installed on the FRP surface at midheight for all the tubes. The SGs were located at the same position as the OFBGs, but distant radially as they were mounted outside the FRP jacket instead of built-in like the OFBGs (see Figure 1). Two linear variable displacement transducers (LVDTs) measured the axial shortening of the specimens. The optical fiber jumpers from the embedded OFBGs were connected to an optical demodulator device (ZX-FP-C16) that recorded all the WS. A high definition camera was used to capture the buckling deformation in the inner tube. The SGs, LVDTs, and the load cell were connected to a data acquisition device (DH3820) (see Figure 3). Although the experiment was conducted at a lab, the climatic condition was not actually at constant temperature. Temperature variation that could affect the WS of the built-in OFBGs was observed during loading. Some precautions were taken for each test specimen by using a stress-free specimen, which served as temperature compensation. The same optical demodulator device collected the WS from the stress-free specimen. The specimens were loaded until failure to investigate their mechanical and self-sensing performance at every stage if possible. The hybrid DSTC fails at the rupture of the outer FRP tube, whereas the BFST-DSTC fails at the rupture of the outer steel skin. A 15 kN preload followed by a displacement control rate of 0.3 mm per minute defined the loading procedure for each specimen.

## 4. Results and Discussions

### 4.1. Mechanical Performance and Behavior of the Proposed BFST-DSTC

#### 4.1.1. Failure and Damage Mechanisms

**Figure 4:**
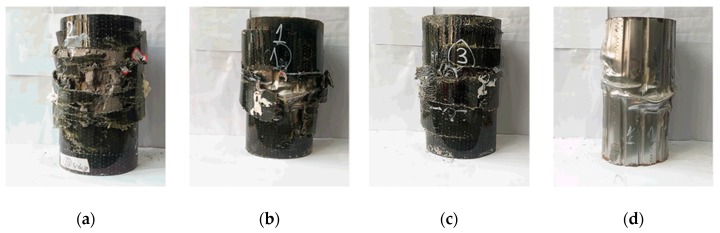
Failure mechanism: (**a**) Abrupt failure of hybrid DSTC; (**b**) initial stage of progressive failure for BFST-DSTC; (**c**) final stage of progressive failure for BFST-DSTC; (**d**) outward buckling and rupture of the outer steel layer in BFST-DSTC.

Generally, the failure mechanism of all the BFST-DSTC specimens includes the rupture of the outer FRP jackets. Steel-adhesive interfacial debonding occurred before the rupture of the outer jacket, characterized by several epoxy sounds. The BFST-DSTCs showed a progressive rupture of the outer FRP jacket (Figure 4b,c), characterized by a gradual decrease in the load capacity. This is due to different factors such as the presence of the steel layer, punched-in pattern, and the use of BFRP. The outer FRP jacket failure started at the mid-height of the specimen (see Figure 4), and then extended towards the ends. The concrete expansion after the initial failure resulted in the outward buckling deformation and rupture of the outer steel layer (see Figure 4d). For comparison, the hybrid DSTC showed a sudden failure at the ultimate load that is depicted in Figure 4a.

#### 4.1.2. Load-Carrying Capacity and Confinement Ratio

The load-carrying capacity and confinement ratio define the strength for FRP-based confined concrete column at the rupture of the FRP. Table 3 highlights the mechanical performance results of each scaled specimen regarding yield load (*P*_y_), load-carrying capacity (*P*_f_), confinement ratio (*ε*_cc_*/ε*_co_), and confinement efficiency (*f*_cc_*/f*_co_). *f*_co_ and *ε*_co_ are the compressive strength and corresponding strain of the unconfined concrete, respectively. *f*_cc_ and *ε*_cc_ are the maximum compressive strength and corresponding strain of the confined concrete. The BFST-DSTC with punched-in pattern showed higher load capacity due to the additional confinement force from the outer punched-in steel layer and inner FRP jacket compared with the hybrid DSTC and the normal BFST-DSTC. The confinement efficiency and confinement ratio were enhanced for all the BFST-DSTC, except for BFST-DSTC1, which could be due to the low confinement efficiency of the two outer FRP layers. The cost-benefit of the proposed system was defined by the highly enhanced ratio of the confinement efficiency up to 40% when three layers of inner FRP jacket and a skinny punched-in steel layer were adopted in the design. The enhanced ratio in Table 3 is the rapport between the confinement efficiency of each specimen with the reference hybrid DSTC. The *P*_y_ of the BFST-DSTC was also enhanced slightly.

#### 4.1.3. Axial Stress–Strain Behavior

**Figure 5:**
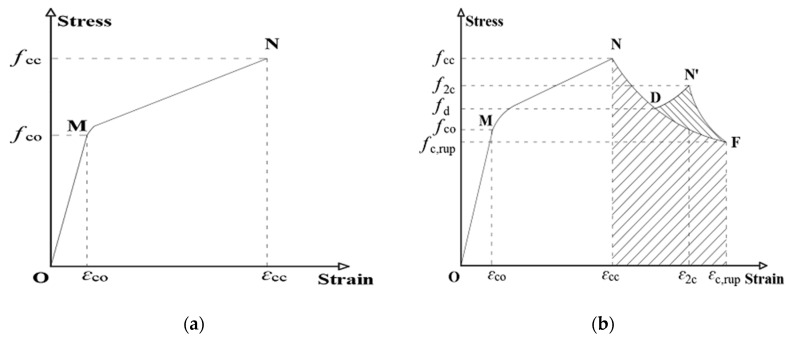
General stress–strain behavior of: (**a**) Hybrid DSTC; (**b**) BFST-DSTC.

The differences in the failure mechanisms of the hybrid DSTC and BFST-DSTC result in distinct changes in the stress–strain behavior. Figure 5a,b stand for the general axial stress–strain behavior of concrete inside the hybrid DSTC and the BFST-DSTC, respectively. The hybrid DSTC has piecewise linear segments separated by a short transition before reaching *f*_cc_. The first segment (OM) ends at the yield point of the inner steel tube. The second linear segment (MN) starts from the full activation to the rupture of the FRP tube, since the strain reading from the OFBGs in the FRP tube is quite small and unstable at the OM part. The stress–strain behavior of the BFST-DSTC (Figure 5b) generally consists of three main sections. First, an ascending piecewise linear (OMN) part with a medium transition. The first linear segment (OM) ends when the inner and outer steel yield. The inner and outer FRP jackets become gradually active in the transition stage. The full activation of both inner and outer FRP jackets to the outer FRP rupture point characterized the second linear segment. The inner FRP jacket ruptured either during the strengthening stage or after the peak load. Unlike the hybrid DSTC, a gradual descending section or “initial failure” (curve ND) (the second section) exists. It starts from the initial rupture of the outer FRP jacket to a stress level (*f*_d_) that is still superior to the yield point (*f*_co_) of the concrete inside the BFST-DSTC. A gradual descent of the loading capacity due to the slow attenuation of the FRP confinement is observed. At last, a residual section, (curve DF or DN’F) depending mainly on the parameters of the inner BFST, will be further explored in future works. A second ascending segment (DN’) with post-peak stress (*f*_2c_) and a degradation part (curve N’F) characterize the residual section for specimens with NSC. For specimens with HSC (curve DF), it decreases progressively until reaching stress equivalent to *f*_c,rup_. The corresponding strain for each specific stress is also plotted in Figure 5. 

### 4.2. Self-Sensing Performance Analysis Based on WS from the Outer Tube

**Figure 6:**
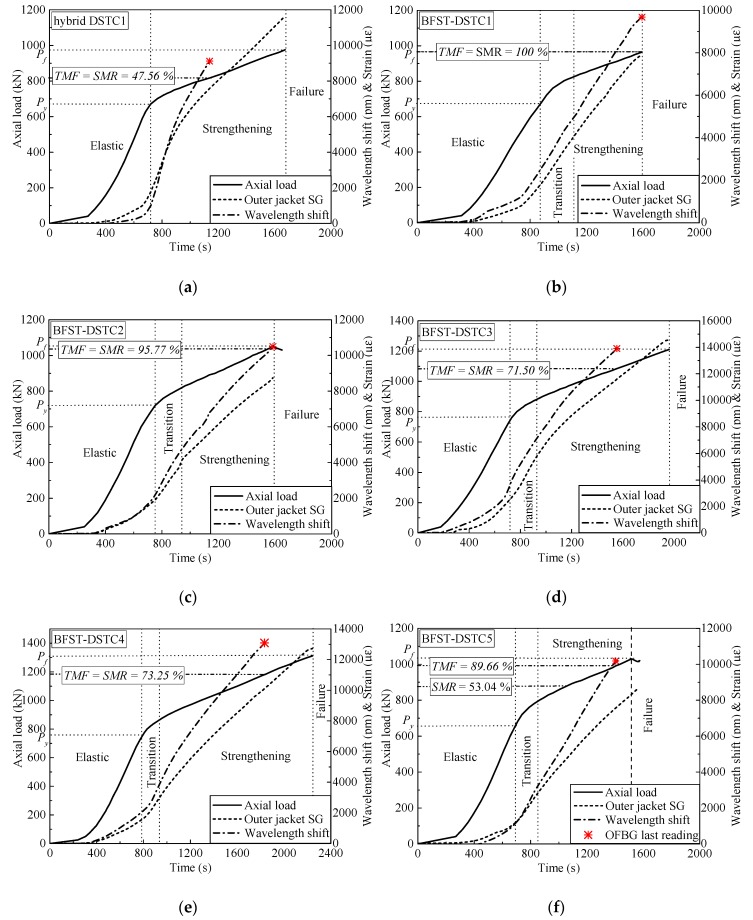
Hoop strain self-sensing performance of the outer tube for specimens with normal strength (concrete) NSC: (**a**) Hybrid DSTC1; (**b**) BFST-DSTC1; (**c**) BFST-DSTC2; (**d**) BFST-DSTC3; (**e**) BFST-DSTC4; (**f**) BFST-DSTC5. TMF: Total measurement from optical fiber Bragg grating sensors (OFBGs), SMR: Strain measurement range, * OFBGs’ last reading.

**Figure 7:**
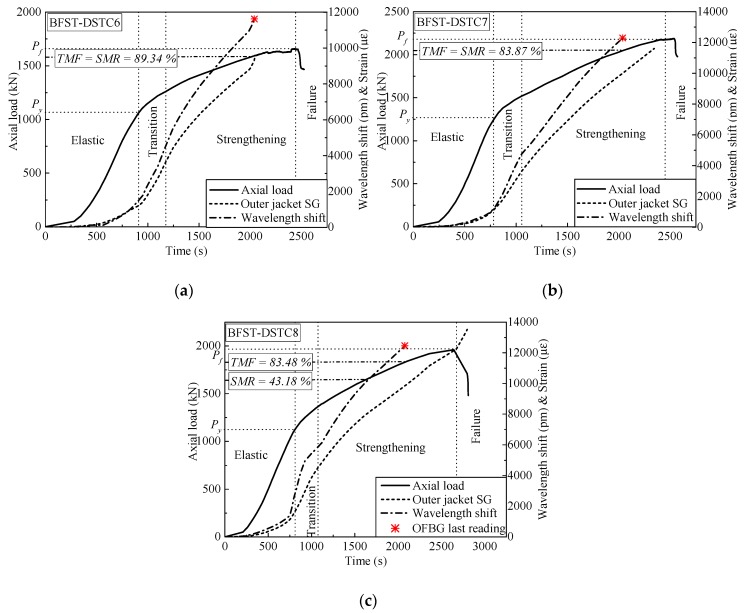
Hoop strain self-sensing performance of the outer tube for specimens with high-strength concrete (HSC): (**a**) BFST-DSTC6; (**b**) BFST-DSTC7; (**c**) BFST-DSTC8. TMF: Total measurement from OFBGs, SMR: Strain measurement range, * OFBGs last reading.

**Figure 8:**
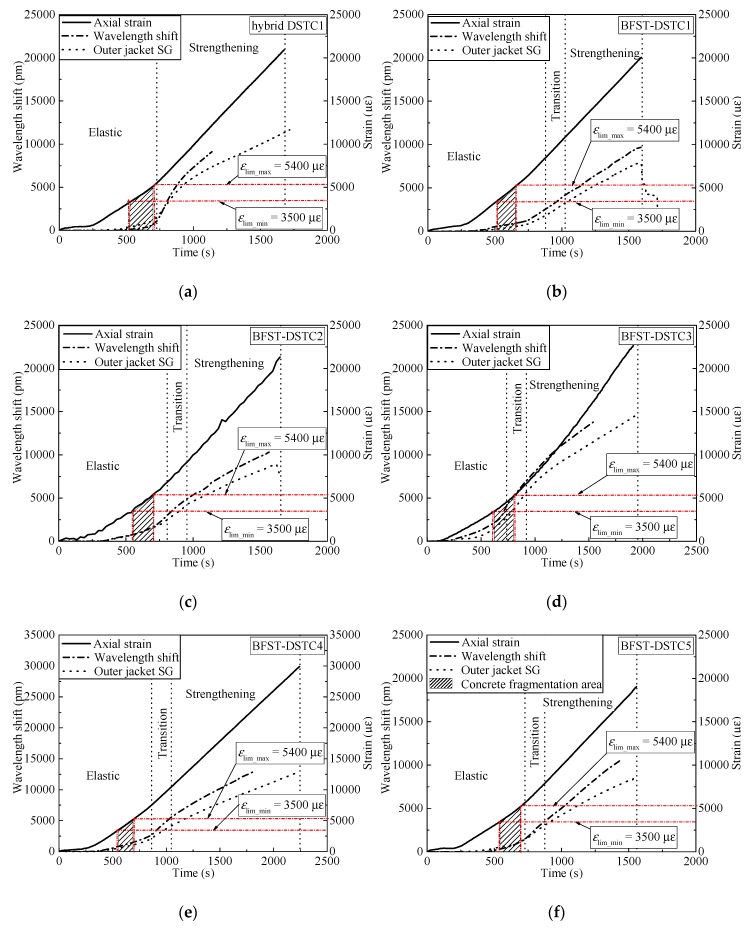

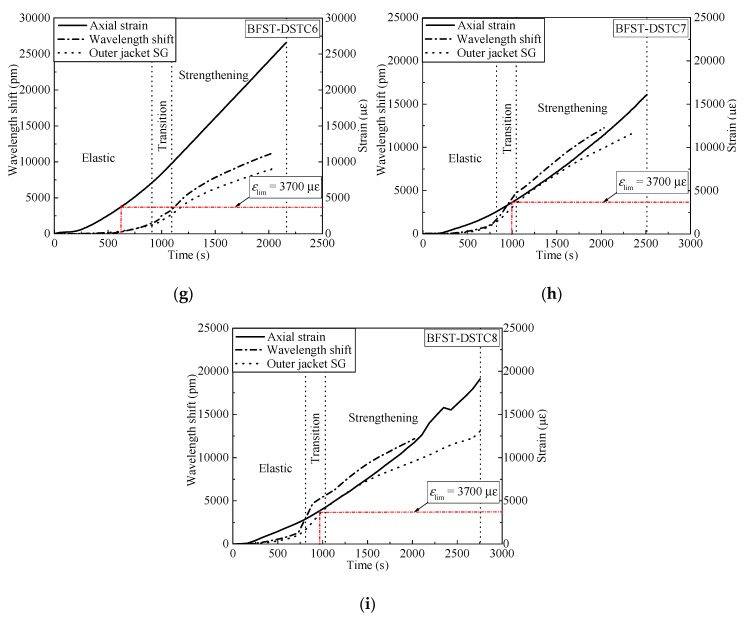
Axial strain monitoring based on outer tube wavelength shift (WS) for specimens: (**a**) hybrid DSTC1; (**b**) BFST-DSTC1; (**c**) BFST-DSTC2; (**d**) BFST-DSTC3; (**e**) BFST-DSTC4; (**f**) BFST-DSTC5; (**g**) BFST-DSTC6; (**h**) BFST-DSTC7; (**i**) BFST-DSTC8.

The self-sensing performance of the built-in OFBGs from the outer tube is analyzed in this section. The WS along with the SG curves are used to describe the deformation occurring in the column. Hereafter, the strain readings from the hoop SGs were considered as reference due to the wide application of SGs in measuring the hoop strain of FRP tubes in FRP-based confined concrete columns. Figure 6 and Figure 7 provide the relationship between the WS, axial load, and hoop strain from the SGs for specimens with NSC and HSC, respectively. The WS of the built-in OFBGs was calculated from Equation (5), due to temperature effect compensation. The inconsistency between the SG and the OFBGs readings is due to the difference in their respective radial position. Assuming that the good interfacial bonding between concrete and the outer tube exists, Figure 8 plots the typical variation of the WS and SG, as well as axial strain versus time. The method of Pham et al. [9] calculated the maximum usable strain limit (*ɛ*_lim_) for the confined concrete, which gave a lower value than the recommended value (1%, equivalent to 10,000 *με*) in ACI 440.2R-08 [37]. The minimum (*ε*_lim_min_ = 3500 *με*) and maximum (*ε*_lim_max_ = 5400 *με*) defined the range of the usable strain limit for specimens with NSC, while the average of *ε*_lim_ was represented for the specimen with HSC. The nominal axial strain was the average axial shortening from LVDTs to the overall height. The self-sensing included only the piecewise linear segments of each respective specimen. The WS and the SG shared a tiny slope at the commencement with an axial load below 200 kN and 500 kN for the specimen with NSC and HSC, respectively. All specimens were healthy, and there was no micro-damage observed yet. Before the steel yielded, the slopes of the WS and SG curves for all specimens increased slowly with the hoop strain around 2000 *με,* except for BFST-DSTC3. According to Figure 8, all specimens reached the *ε*_lim_min_. The concrete core deformation accompanied with the outer steel layer confinement activation might have caused the increase of the WS slopes before the yielding point. The *ε*_lim_ for all specimens could be effectively monitored based on the relationship between the WS and the axial strain. The sharp changes of the WS curves were due to concrete expansion and the steel yielding. However, the WS curves were still reliable for assessing the strain developed in the outer FRP jacket. The increase in the gap between the SG and WS was considered as a warning sign of the full activation of the outer FRP jacket. The hoop strain values were below 4000 *με,* except for the BFST-DSTC3 (Figure 6d), which reached around 6000 *με*.

In the strengthening stage, the hoop strain developed in the outer FRP jacket is essential to monitor due to its brittle failure. The measurement ranges of the built-in OFBGs are introduced to investigate their self-sensing performance. Table 4 provides the strain measurement range (SMR) when the error between the OFBGs and SG are included in the interval of ±2.5% (i,e., −2.5% < SMR < +2.5%), and the total measurement from the OFBGs (TMF). The SMR and TMF were obtained by dividing their corresponding loads by the difference between the ultimate load (*P*_f_) and the yield load (*P*_y_). All the BFST-DSTCs have a minimum TMF of 71.5%. Within these values, the measured strain of the FRP jacket reached 8000 *με* to 10,000 *με* for specimens with NSC (see Figure 6b–f), and more than 10,000 *με* for specimens with HSC (Figure 7a–c). These values are remarkable and wide enough to monitor the usable and limit strains of the outer FRP jacket (see Figure 8b–i). The FRP tube in hybrid DSTC1 had a TMF inferior to 50%, with a total measured strain less than 7500 *με* (Figure 6a). The SMR for the BFST-DSTC was more than 50% compared to that of the hybrid DSTC (47.48%). Only specimen BFST-DSTC8 (Figure 7b) had an SMR smaller than that of the hybrid DSTC1. The strain developed in the outer FRP jacket had already reached around 8000 *με*, except for specimen BFST-DSTC5 (Figure 6f).

**Figure 9:**
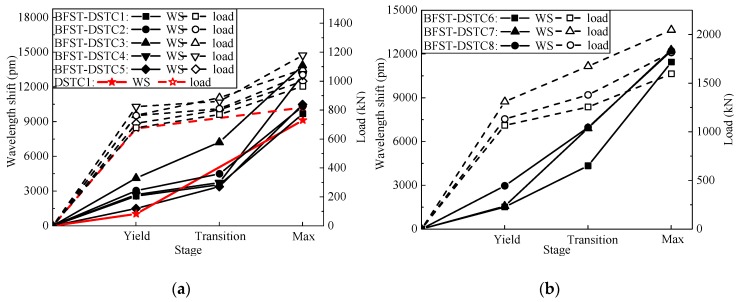
Relationship between the outer tubes’ WS and the load at each stage: (**a**) Specimens with NSC; (**b**) specimens with HSC.

The relationship between the axial load and the WS of the built-in OFBGs from the outer tube is established here. Figure 9 illustrates the relationship between the axial load and the WS versus time (represented as each loading stage). The “Max.” stage corresponds to the load where the last reading from the WS was gathered. The WS and the load were divergent in the elastic stage. At the transition part, a convergent relationship existed for all specimens until the end. A linear relationship existed between the load and the WS for each piecewise linear segment. Thus, the loading process could be well monitored by the WS from the outer FRP jacket. 

### 4.3. Self-Sensing Performance Analysis Based on WS from the Inner Tube

**Figure 10:**
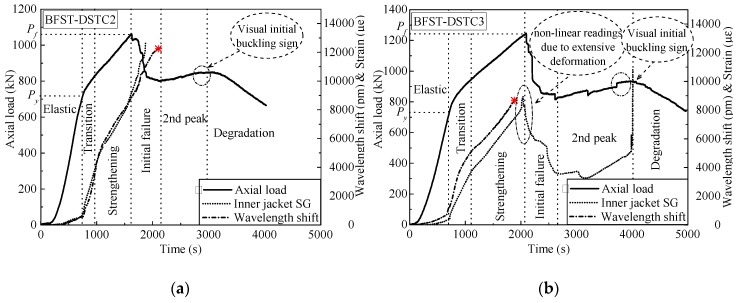

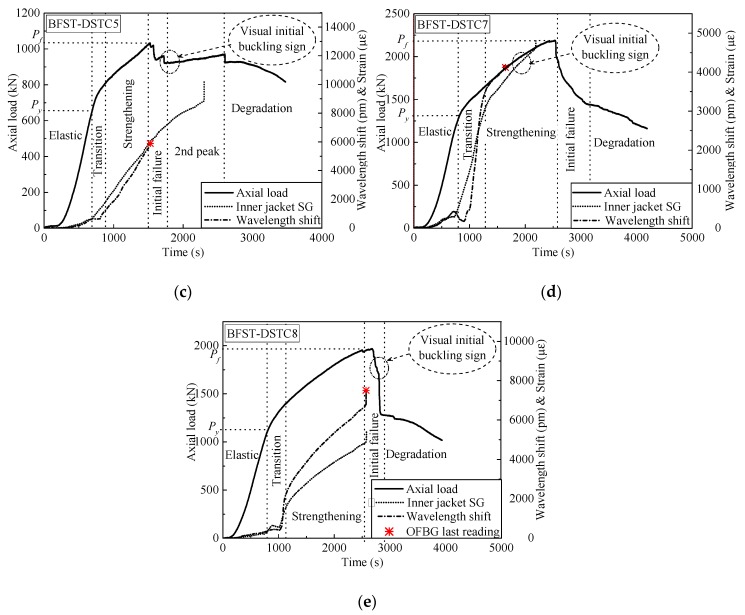
Inner tube self-sensing response based on accumulated WS for specimen: (**a**) BFST-DSTC2; (**b**) BFST-DSTC3; (**c**) BFST-DSTC5; (**d**) BFST-DSTC7; (**e**) BFST-DSTC8.

**Figure 11:**
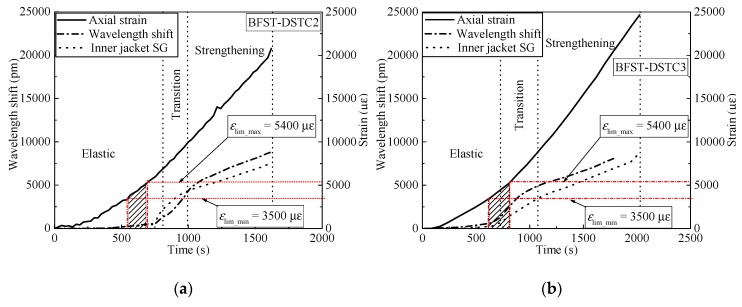

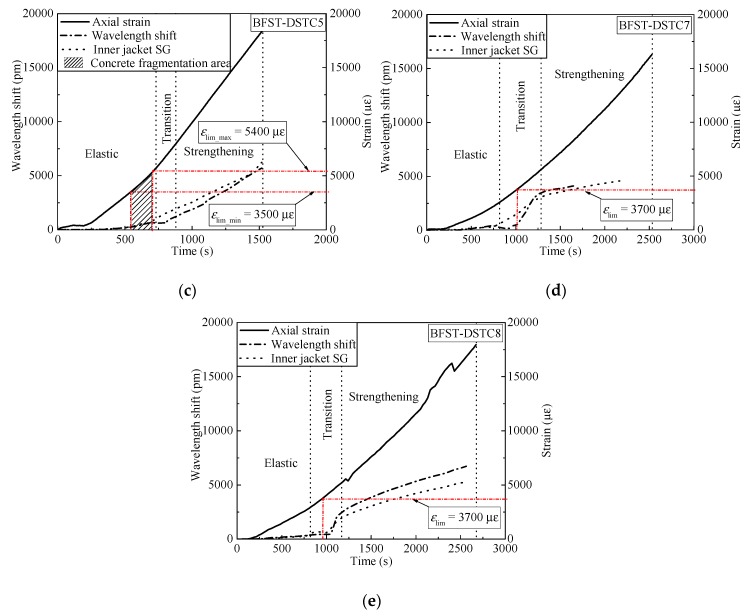
Axial strain monitoring based on the inner tube WS for specimens: (**a**) BFST-DSTC2; (**b**) BFST-DSTC3; (**c**) BFST-DSTC5; (**d**) BFST-DSTC7; (**e**) BFST-DSTC8.

This section discusses the self-sensing performance of the built-in OFBGs in the inner tube to detect the deformation of the column with the increase of load. The WS curve for each specimen has been compared with the SG curve. Figure 10 plots the WS response from the embedded OFBGs in the inner tube versus time, axial load, and hoop strain from the SGs. Figure 11 depicts the typical variation of the inner WS and SG, as well as axial strain versus time, to investigate the concrete core interaction. Similarly to the outer tube, the WS for each specimen was calculated by deducing the effect of temperature using Equation (5). In the elastic stage, slow rising tendencies were observed from the responses of the SG and the OFBGs. The healthy condition of the inner tube and the BFST-DSTC design are the reason behind such observation. The maximum usable strain limit (*ε*_lim_) of the confined concrete fell in the elastic stage for specimen BFST-DSTC2, BFST-DSTC3, and BFST-DSTC5 (Figure 11a–c). The relationship between the axial and the WS for these specimens could monitor well the *ε*_lim_. In the transition part, the inner steel yielded, and the inner FRP jacket started to activate gradually. A sudden drop of the WS in specimens with HSC (Figure 10d,e, i.e., BFST-DSTC7 and BFST-DSTC8) was noticed at the start of their transition stage. The *ε*_lim_ (see Figure 11d,e) fell in the transition branch, which was due to the brittle failure of the HSC. However, the WS regained its growth tendency with the SG due to the radial pressure from the concrete core to the inner FRP jacket. Specimens with NSC showed a gradual ascending of the WS and SG, whereas specimens with HSC displayed abrupt rising tendencies.

In the strengthening stage, the self-sensing performance depended on the parameters of the inner BFST. Here, how far the inner BFST can provide data for its self-damage analysis is the focus. The measurement ranges of each specimen were analyzed based on the error between the readings of the built-in OFBGs and the SG. The WS for BFST-DSTC2 (Figure 10a) ended at the final of the initial failure stage, reaching a total value up to 12,000 pm. The inner FRP jacket is assumed to fail at this stage, so the OFBGs can be used to detect the failure of the BFST-DSTC. The high deformation developed in the inner steel tube caused the difference between the WS and SG tendencies from the initial failure. Visual observation from the high definition camera showed that the initial buckling signs appeared during the second peak stage. Figure 10b demonstrates that the buckling deformation of the BFST-DSTC3 appeared in the later stage of the second peak. However, the WS terminated before the expected time with a strain value superior to 7000 *με*. In the BFST-DSTC5 (Figure 10c), the WS curve ended at the start of the initial failure. The visual buckling sign was observed at the end of the initial failure stage. The WS terminated before specimens reached the peak load. Plastic deformation occurred during the strengthening stage due to the insufficient radial pressure from the inner FRP jacket, which resulted in the early buckling deformation. The WS data from the inner BFST are enough to monitor the condition of the inner tube as well as the whole system before any significant damage occurs. 

**Figure 12:**
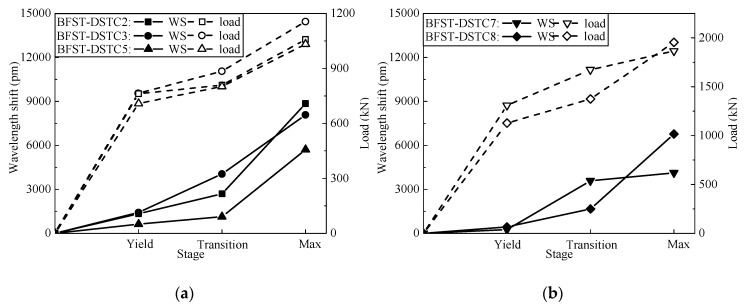
Relationship between the outer tube’s WS and the load at each stage: (**a**) Specimens with NSC; (**b**) Specimens with HSC.

The WS of the built-in OFBGs from the inner tube could monitor the axial load history of each specimen. A relationship can be established to support the results from the built-in OFBGs of the outer tube if the latter is broken before reaching the maximum load. Figure 12 shows the relationship between the load and the WS from the inner tube for each stage. The WS and the load were also divergent in the elastic stage. As the load increased, a proportional relationship existed between the WS and the load after the yield. Both Figure 12a,b demonstrate that the increase of the load-carrying capacity is due to outer FRP jacket. 

## 5. Conclusions

This paper presents the experimental investigation of the self-sensing performance for a proposed smart BFST-DSTC with built-in OFBGs under axial compression. The conclusions of this paper are listed as follows:

- With the built-in concept, an innovative FRP-based confined concrete system with self-sensing ability is easy to develop and cost-effective. The proposed smart BFST-DSTC shows potential mechanical benefits to overtake the hybrid DSTC as a modern form of structural columns. The additional inner FRP layer and outer steel skin with punched-in patterns improve the loading capacity, the confinement ratio, and the confinement efficiency compared with the hybrid DSTC.
- The OFBGs embedded in the outer tube satisfied the self-monitoring requirements of the strain condition in the proposed smart BFST-DSTC and hybrid DSTC within their service limit. Specimens with punched-in outer steel had a SMR and TMF beyond 70% of the strengthening load. These outcomes are wide enough to detect minor and major deformations of the BFST-DSTC.
- The embedded OFBGs monitor the hoop strain of the inner and outer FRP jackets and provide early failure warning for the inner tube. The WS from the inner FRP jacket can help for further damage evaluation of the BFST-DSTC in the post-peak stage.
- The load and WS curves from the inner and outer tubes showed good linear relation at each piecewise segment. The WS of the built-in OFBGs in both tubes can monitor the axial load history of the FRP-confined concrete.

The findings in this paper would be a useful contribution to the R&D of smart civil infrastructures. However, further investigation and optimization design are still needed to improve the self-sensing performance of the smart BFST-DSTC developed in this study.

## Figures and Tables

**Table 1:** Smart column details.

Specimen no.	Inner Tube				Outer Tube					Concrete Strength [MPa]
	*t_is_*^3^ (mm)	*d_is_*^4^ (mm)	*n_i_* ^5^	Type	*t_os_*^6^ (mm)	Steel Layer Type	*d_os_*^7^ (mm)	*n_o_* ^8^	Type	
Hybrid DSTC1	4.0	100	0	NS ^1^	0	N/A	N/A	3	NS ^1^	20.02
BFST-DSTC1	4.0	100	2	Smart ^2^	0.7	Punched-in	150	2	Smart	20.02
BFST-DSTC2	4.0	100	2	Smart	0.7	Punched-in	150	3	Smart	20.02
BFST-DSTC3	4.0	100	3	Smart	0.7	Punched-in	150	3	Smart	20.02
BFST-DSTC4	4.0	100	3	Smart ^2^	1.2	Punched-in	150	3	Smart	20.02
BFST-DSTC5	4.0	100	2	Smart	0.7	Normal	150	3	Smart	20.02
Hybrid DSTC2	4.0	100	0	NS ^1^	0	N/A	N/A	3	NS ^1^	60.55
BFST-DSTC6	4.0	100	2	Smart ^2^	0.7	Punched-in	150	6	Smart	60.55
BFST-DSTC7	4.0	100	3	Smart	0.7	Punched-in	150	9	Smart	60.55
BFST-DSTC8	4.0	100	2	Smart	0.7	Normal	150	9	Smart	60.55

^1^ Not smart; ^2^ optical fiber jumper was broken before testing; ^3^ and ^4^ inner steel thickness and diameter; ^6^ and ^7^ outer steel thickness and diameter; ^5^ and ^8^ numbers of inner and outer FRP layers.

**Table 2:** Material properties of steel.

Steel Thickness [mm]	Yield Strength [MPa]	Tensile Modulus [GPa]	Ultimate Stress [MPa]
4.0	270.64	191.0	335
6.0	260.15	189.0	410
0.7	196.19	189.5	313.12
1.2	195	189.5	313.12

**Table 3:** Mechanical test results.

Specimen no.	*P*_y_ [kN]	*P*_f_ [kN]	*f*_cc_/*f*_co_	Enhanced Ratio	*ɛ*_cc_/*ɛ*_co_
Hybrid DSTC1	673.4	975.6	1.31	1	10.45
BFST-DSTC1	679.3	964.4	1.23	0.94	10.03
BFST-DSTC2	761.7	1061.7	1.42	1.08	10.42
BFST-DSTC3	764.3	1211.7	1.83	1.42	17.16
BFST-DSTC4	823.7	1312.3	1.43	1.09	14.96
BFST-DSTC5	708.5	1033.1	1.36	1.04	9.18
Hybrid DSTC2	1022.1	1505.4	1.59	1	9.60
BFST-DSTC6	1062.7	1657.1	1.45	0.91	11.89
BFST-DSTC7	1309.4	2189.6	2.30	1.45	18.33
BFST-DSTC8	1127.9	1968.8	1.87	1.18	16.02

**Table 4:** TMF, SMR, and error for the outer tubes.

Specimen	SMR (%)	Error (%)	TMF (%)
Hybrid DSTC1	47.56	+1.11	47.56
BFST-DSTC1	100.00	+1.57	100.00
BFST-DSTC2	96.01	−0.31	96.01
BFST-DSTC3	71.50	−0.94	71.50
BFST-DSTC4	73.25	−1.75	73.25
BFST-DSTC5	53.04	+2.37	89.66
BFST-DSTC6	89.34	+0.12	89.34
BFST-DSTC7	83.87	+1.37	83.87
BFST-DSTC8	43.18	+1.98	83.48

## Abbreviations

ASTM	American standard test method
BFRP	Basalt fiber reinforced polymer
BFST	Basalt FRP-steel tube
CFFT	Concrete-filled FRP tube
CFST	Concrete-filled steel tube
DSTC	Double-skin tubular column
*ɛ*_cc_	Compressive strain of confined concrete
*ɛ*_co_	Compressive strain of unconfined concrete
*ɛ*_lim_	Average strain limit value of concrete in specimens with HSC
*f*_2c_	Compressive strength at the 2nd ascending segment at the post-peak
*f*_cc_	Compressive strength of confined concrete
*f*_co_	Compressive strength of unconfined concrete
*f*_d_	Compressive strength of confined concrete after the rupture of the FRP
FRP	Fiber-reinforced polymer
GFRP	Glass fiber reinforced polymer
HSC	High strength concrete
LVDTs	Linear variable displacement transducers
NSC	Normal strength concrete
OFBGs	Optical fiber Bragg grating sensor
OFS	Optical fiber sensors
*P*_f_	Ultimate load/Maximum load capacity of the column
*P*_y_	Yield load/load at the yield point
SG	Electric strain gauge
SMR	Strain measurement range of the built-in OFBGs with error between OFBGs and SG ±2.5%
TMF	Total measurement from OFBGs
UV	Ultraviolet
WS	Wavelength shift
*ε*_lim_max_	Maximum strain limit value of concrete in specimens with NSC
*ε*_lim_min_	Minimum strain limit value of concrete in specimens with NSC
